# First Report and Comprehensive Risk Index of *bla*_IMP-1_-Harboring *Brucella anthropi* in Municipal Wastewater-Irrigated Soil

**DOI:** 10.3390/microorganisms14030688

**Published:** 2026-03-18

**Authors:** Ling Zhao, Yanhao Wu, Runze Xu, Xuewen Li

**Affiliations:** Department of Environment and Health, School of Public Health, Cheeloo College of Medicine, Shandong University, Jinan 250012, China; sddxlanezhao@163.com (L.Z.); 15762466880@163.com (Y.W.); 13954868066@163.com (R.X.)

**Keywords:** wastewater treatment plant, *Brucella anthropi*, carbapenemases, plasmids, horizontal gene transfer

## Abstract

*Brucella anthropi* is an emerging opportunistic pathogen characterized by intrinsic resistance to most β-lactams. However, the acquisition of carbapenem resistance in this species has rarely been documented in environmental, animal, or clinical settings. In this study, a multidrug-resistant strain, SBA01, was isolated from wastewater-irrigated soil. SBA01 exhibited phenotypic resistance to carbapenems and colistin, the latter being independent of *mcr* genes. Genomic analysis localized *bla*_IMP-1_ on a stable 21 kb plasmid maintained by a Type II toxin–antitoxin system. While non-self-transmissible, this plasmid was mobilized to *Escherichia coli* and *Klebsiella pneumoniae* via an unclassified 50 kb helper plasmid. Additionally, a 217 kb prophage-bearing megaplasmid was identified, enhancing genomic plasticity. Genomic screening identified 32 putative virulence determinants, including markers associated with host interaction. Risk profiling indicated an elevated hazard index for SBA01, driven by the convergence of multidrug resistance, cryptic mobilization capacity, and opportunistic survival traits. These findings position B. anthropi as a resilient environmental reservoir for clinically relevant carbapenemases. Expanding surveillance frameworks to include such adaptive hosts is necessary to better evaluate potential occupational exposures at the wastewater–soil interface.

## 1. Introduction

Recent clinical surveillance has indicated an increasing incidence of *Brucella anthropi* (formerly *Ochrobactrum anthropi*) infections, identifying it as an emerging opportunistic pathogen [1,2,3,4]. Although taxonomic reclassification aligning this organism with the highly virulent *B. melitensis* raised concerns regarding its pathogenicity, comparative genomics has revealed that *B. anthropi* lacks the signature intracellular virulence determinants found in classical *Brucella* species [5]. Unlike niche-restricted *Brucella* lineages, *B. anthropi* exhibits exceptional genomic plasticity and competence for mobile genetic elements [6,7], as exemplified by the multi-replicon structure of the reference strain ATCC 49188 [8]. Consequently, the absence of classical virulence factors is likely counterbalanced by the rapid acquisition of multidrug resistance and secondary virulence modules, thereby accelerating the evolution of *B. anthropi* into a critical pathogenic threat.

Clinically, this genomic versatility exacerbates the therapeutic challenges posed by the intrinsic resistance of the bacterium. *B. anthropi* possesses intrinsic resistance to most β-lactams, resulting in limited therapeutic options that are easily compromised by the acquisition of secondary resistance traits [9,10]. Notably, in 2022, a report documented an IMP-producing *B. anthropi* strain from a carbapenem-resistant infection [11], indicating that this organism has transitioned from an environmental saprophyte to a reservoir of last-resort antibiotic resistance. Crucially, such clinical emergence likely represents only the endpoint of an evolutionary process, and the genetic assembly of these high-risk phenotypes likely occurs earlier in anthropogenic environments characterized by intense selection pressure.

Wastewater Treatment Plants (WWTPs) serve as critical repositories for antibiotic resistance genes (ARGs) and pathogens, creating continuous selection pressure that drives microbial evolution [12,13]. Although current biosecurity protocols often focus on treated effluents, the subsequent application of reclaimed water for irrigation introduces a direct interface between these reservoirs and agricultural soils [14]. In this complex matrix, the convergence of residual antibiotics, heavy metals, and high bacterial density facilitates horizontal gene transfer (HGT) [15], transforming soil microflora into persistent reservoirs of multidrug resistance. Crucially, evidence suggests that pathogens, such as *B. anthropi*, do not remain static in this environment and can be aerosolized from contaminated soil, particularly during irrigation and tillage operations [16]. This bioaerosol pathway establishes a plausible transmission route to the respiratory tract of agricultural and sanitation workers, potentially facilitating colonization and subsequent dissemination into broader community settings [17].

In the present study, a *bla*_IMP-1_-carrying *B. anthropi* strain (SBA01) was isolated from municipal wastewater-irrigated soil. This isolation occurred in 2019, predating the major clinical reports of this specific phenotype and suggesting an earlier environmental origin. By characterizing SBA01, the molecular mechanisms underlying its resistance, stability, and capacity for transspecies dissemination were investigated. Furthermore, a comprehensive risk index was used to quantify the pathogenicity and mobility of this lineage, providing a practical basis for controlling its environmental and occupational transmission.

## 2. Materials and Methods

### 2.1. Sample Collection, Isolation, and Identification

Sampling was conducted on 24 January 2019, at a wastewater treatment plant (WWTP) in Jinan, Shandong Province, Eastern China (116°57′14.07″ E, 36°41′38.86″ N). The sampled green spaces have a documented history of continuous irrigation with effluent which represents the final discharge point into the receiving river ecosystem since approximately 2007, at a frequency of one to two times per week. Four composite superficial soil samples (depth 0–10 cm) were collected. For each of the four designated sampling plots (standardized to approximately 10 × 10 m), a five-point sampling strategy was strictly employed, wherein five distinct core subsamples (from the four corners and the center) were pooled to generate a single representative composite sample [18]. At least 30 g of soil samples were collected in sterile plastic bottles, placed in a cooler box at 4 °C, and transported to the laboratory within 12 h for immediate processing. The soil samples were subjected to a pre-enrichment step in brain–heart infusion broth, which was performed overnight before application to the plates. The enriched solutions (100 µL) were plated on MacConkey agar (Oxoid, Basingstoke, UK) supplemented with 2 mg/L of meropenem (Meilun, Dalian, China) to isolate potential carbapenem-resistant isolates [19]. Species identification was performed using matrix-assisted laser desorption/ionization time-of-flight mass spectrometry (Bruker, Billerica, MA, USA) [20].

### 2.2. Genomic DNA Sequencing and Bioinformatics Analysis

Genomic DNA of the donor strain SBA01 was extracted and purified using the Gentra Puregene Yeast/Bact. Kit (Qiagen, Hilden, Germany). To resolve complex plasmid architectures, a hybrid sequencing strategy was employed. Short-read sequencing was performed on the Illumina NovaSeq 6000 platform (San Diego, CA, USA) with 150 bp paired-end reads, while long-read sequencing was executed on the Oxford Nanopore Technologies MinION platform (Oxford, UK) [20]. Hybrid de novo assembly was conducted using Unicycler v0.4.8 in ‘--bold’ mode to achieve complete circularization [21]. Structural closure of the chromosome and plasmids was confirmed by identifying terminal overlapping sequences at unitig extremities.

Definitive taxonomic classification of SBA01 was confirmed by calculating the Average Nucleotide Identity (ANI) against the *B. anthropi* reference genome (ATCC 49188T) using FastANI v1.34, applying a species-delineation threshold of >95%. Genome annotation was performed via the Bacterial and Viral Bioinformatics Resource Center (BV-BRC) server (Chicago, IL, USA). Resistome and virulome profiles were characterized using the BV-BRC Comprehensive Genome Analysis service, incorporating CARD v3.1.2 (identity ≥ 95%, coverage ≥ 95%) and VFDB v3.3.0 (E-value < 1 × 10^−10^, identity > 80%) [22]. To pre-evaluate the mobilization potential of the identified extrachromosomal elements, potential origins of transfer (oriT) and mobilization modules were profiled using OriTDB (https://bioinfo-mml.sjtu.edu.cn/oriTDB2/oriTfinder.php; accessed on 1 February 2026) [23], and plasmid incompatibility (Inc) typing was performed via PlasmidFinder v2.0.1. Integrated prophages were predicted using the PHASTER web server, retaining only ‘intact’ regions (score > 90) [24]. The genetic-environment comparison chart was created using Easyfig v2.2.2 [25].

For comparative phylogenomic analysis, *B. anthropi* genomic sequences were retrieved from the BV-BRC database. Quality control was enforced using stringent inclusion criteria: CheckM completeness ≥ 95%, contamination ≤ 2%, contig L50 ≤ 20, and N50 ≥ 150,000 bp. This rigorous filtering yielded 72 high-quality genomes (accession IDs and metadata are detailed in Table S7). A Neighbor-Joining (NJ) tree was constructed utilizing the BV-BRC Bacterial Genome Tree service. Specifically, whole-genome single-nucleotide polymorphisms (SNPs) were extracted from aligned global conserved protein families (PATRIC GFams), with branch stability evaluated via 100 bootstrap replicates. Clade assignments were established based on terminal branching architecture, where a “mixed-source lineage” was strictly defined as a localized cluster characterized by the direct interspersion of environmental and anthropogenic terminal nodes. The final phylogeny was visualized and annotated using iTOL v6 [26].

To elucidate the fundamental genetic basis of the intrinsic colistin resistance, a targeted comparative analysis was executed within the R v4.4.1 programming environment. Initially, a curated theoretical panel of candidate genes canonically associated with polymyxin tolerance and envelope regulation was established. Utilizing the annotated protein sequence files (.faa), an orthology-based intersection was performed to extract the shared (core) genetic repertoire among three strategically selected *B. anthropi* genomes: the target multidrug-resistant strain SBA01, the wild-type reference strain ATCC 49188, and a formally characterized colistin-susceptible clinical isolate, T210003. Utilizing the shared orthologous protein sequences, systematic multiple sequence alignments were conducted to pinpoint amino acid substitutions and structural variations exclusively harbored by SBA01. Special analytical focus was directed toward identifying nonsynonymous mutations within two-component regulatory systems and multidrug efflux pump repressors (e.g., phoP, mexP, and nfxB) [27].

### 2.3. Conjugation Experiments

To fundamentally validate the bioinformatically predicted mobilization machinery, broth mating assays were conducted in independent biological triplicates. *Escherichia coli* J53 (azide-resistant) and *Klebsiella pneumoniae* K19 (exhibiting a high amikacin MIC) served as recipient strains. Robust controls were established: individual donor/recipient cultures were used to confirm the efficacy of the selective media, and a parallel mating mixture treated with DNase I (100 U/mL) was incorporated to definitively rule out natural transformation. Donor and recipient cultures were grown to the logarithmic phase (OD600 ≈ 0.6), mixed at a 1:1 volume ratio, and co-incubated overnight (16 h) at 37 °C without agitation. Following serial dilution, transconjugants were recovered on selective agar supplemented with 2 mg/L imipenem and respective counter-selection antibiotics (sodium azide for J53; amikacin for K19). Conjugation frequencies were subsequently calculated as the number of transconjugant colony-forming units (CFUs) per donor CFU [19].

To elucidate the co-transfer dynamics of the identified extrachromosomal elements, genomic DNA of TBK1 was sequenced on the Illumina NovaSeq 6000 platform. Short-read assembly was performed using Unicycler v0.4.8 in short-read-only mode. The structural integrity of the co-transferred bipartite system was validated by aligning the TBK1 raw reads against the high-quality, circularized reference templates of pSBA01-IMP and pSBA01-con established from the donor strain. This mapping-based verification utilized a stringency threshold of >99.9% sequence identity and 100% breadth of coverage, effectively filtering background recipient chromosomal DNA. Furthermore, the relative plasmid copy number (PCN) in the transconjugant was estimated by normalizing the mean mapping depth of the plasmid-specific contigs against the average chromosomal coverage of the *K. pneumoniae* recipient.

### 2.4. Antimicrobial Susceptibility Testing

The minimum inhibitory concentrations (MICs) of eight antibiotics—meropenem, ertapenem, colistin, ceftazidime/avibactam, tigecycline, cefotaxime, ciprofloxacin, and amikacin (Meilun, Dalian, China)—were determined for the isolates. Routine susceptibility testing was performed using the agar dilution method as recommended by the Clinical and Laboratory Standards Institute (CLSI). However, tigecycline and colistin were evaluated using the broth microdilution method. Specifically for colistin, to prevent drug absorption artifacts, assays were strictly conducted following ISO 20776-1 guidelines utilizing unsupplemented cation-adjusted Mueller–Hinton broth (CAMHB) and untreated polystyrene microtiter plates without the addition of polysorbate 80 [28].

The MIC results were primarily interpreted according to CLSI (2020) breakpoints. Due to the absence of species-specific clinical breakpoints for *B. anthropi*, the MICs for tigecycline and colistin were interpreted utilizing the European Committee on Antimicrobial Susceptibility Testing (EUCAST, version 10.0) breakpoints, with colistin specifically referring to the non-species-related pharmacokinetic/pharmacodynamic (PK/PD) resistance threshold (>2 μg/mL). Quality control was successfully validated using *Escherichia coli* ATCC 25922, with all MICs falling within acceptable standard ranges.

### 2.5. Multidimensional Quantitative Health Risk Index Framework

To comprehensively evaluate the potential health hazards posed by SBA01, a weighted vector-based risk index model was developed that integrated the phenotypic (PRI), genotypic (GRI), and spreading (SRI) risk dimensions.

The *PRI* quantifies clinical treatment challenges. To ensure comparability, the index was standardized against 12 core antibiotic classes prioritized by the WHO (available at https://www.who.int/publications/i/item/9789241515528, accessed on 1 February 2026), including carbapenems, polymyxins, and third-generation cephalosporins. Scores were weighted by clinical hierarchy ($W_{p,i}$): $W_{p,i}=3$ for “last-resort” agents (carbapenems and colistin), $W_{p,i}=2$ for high-priority broad-spectrum agents, and $W_{p,i}=1$ for other standard therapeutic classes. Intrinsic resistance profiles were excluded to avoid score inflation. *PRI* is defined as:

$$PRI=\frac{\sum_{i=1}^{n}(I_{i}\times W_{p,i})}{\sum_{i=1}^{n}W_{p,i}}$$

where $I_{i}$ represents the binary resistance status (1 for non-susceptible and 0 for susceptible). The denominator, mathematically expressed as the sum of all assigned constant weights ($\sum W_{p,i}$), establishes the theoretical maximum score, reflecting a hypothetical pan-resistant profile across all 12 evaluated antibiotic classes.

The *GRI* represents the cumulative hazard of genetic determinants, integrating both ARGs and virulence factors.

$$GRI_{raw}=\sum (W_{ARG}\times N_{ARG})+\sum (W_{VF}\times N_{VF})$$

For ARGs, baseline weights ($W_{ARG}$) were initially mapped to the sequence-level risk ranking framework (ARGrank) defined by Zhang et al. [29]: Rank I (highest risk) = 10; Rank II (high risk) = 5; Rank III (mid risk) = 3; Rank IV (low risk) = 1. However, to account for algorithmic limitations (e.g., environmental masking) in the reference database and ensure conservative risk profiling, a systematic four-tier curatorial criterion was applied: (1) Intrinsic Resistance Exclusion ($W_{ARG}=0$): Chromosomally encoded determinants reflecting the species-specific biological baseline were excluded to negate genomic background noise. (2) Sequence-level Discrepancy Resolution: Determinants with multi-rank annotations across different reference alleles (e.g., *floR* annotated as both Rank I and IV) were strictly categorized by their highest documented rank to ensure a conservative estimation. (3) Manual Escalation for Last-Resort Targets: High-risk plasmid-borne ARGs explicitly compromising WHO ‘Reserve’ category antibiotics (e.g., the carbapenemase), even if unlisted in the initial training set, were phenomenologically escalated to Rank I ($W_{ARG}=10$). (4) Homolog-based Inference for Unmapped Targets: For acquired determinants targeting standard antibiotics lacking direct allele matches, weights were inferred based on the predominating rank of their closest functional homologs (typically Rank IV, $W_{ARG}=1$). The detailed classifications and assigned weights for all identified ARGs are summarized in Table S5. For virulence factors, the following hierarchical criteria were established: Tier 1 ($W_{VF}=5$) for master conserved envelope regulators and structural determinants crucial for extreme environmental resilience and potential host-adaptability (including the *bvrR/S* system and *ricA*), and Tier 2 ($W_{VF}=1$) for general biosynthetic or metabolic components (including *lpx* and *manA*). The complete inventory and tier assignments of VFs are provided in Table S6.

To ensure dimensional consistency, $GRI_{raw}$ was normalized to $GRI_{norm}$ on a scale of [0,1] using a reference maximum value ($GRI_{max}=100$, representative of highly virulent clinical pathogens), this threshold reflects the theoretical upper bound of genetic hazards identified in highly virulent clinical pathogens (e.g., co-occurrence of multiple high-risk ARGs and essential virulence regulators):

$$GRI_{norm}=min(1,\frac{GRI_{raw}}{GRI_{max}})$$

The *SRI* evaluates the potential for horizontal gene transfer (HGT), prioritizing functional biological evidence. The index is defined as the maximum value between experimental observations and bioinformatic predictions. An experimental score ($S_{exp}$) of 1.0 is systematically assigned upon phenotypic confirmation of successful HGT in mating assays.

$$SRI=\max\left(S_{exp},S_{bioinfo}\right)$$

A maximum experimental score ($S_{exp}$) of 1.0 is assigned only upon phenotypic confirmation of successful HGT via mating assays. The bioinformatic score ($S_{bio}$) characterizes the genomic context of the target ARG. Given the absence of standardized replicon-typing profiles for this genus in public databases, plasmid identification relied on the structural resolution of complete, circular extrachromosomal contigs via hybrid assembly. Subsequent mobility classification was determined through functional annotation utilizing OriTDB. $S_{bio}$ was assigned based on the following hierarchy: 0.8 for ARGs localized on self-transmissible plasmids (encoding both a complete T4SS and a relaxase); 0.5 for mobilizable plasmids (harboring relaxases or *oriT* sequences but lacking a functional T4SS); and 0.3 for ARGs associated with IS elements, transposons, or integrons without broader conjugal machinery. In multi-plasmid systems, if multiple plasmids or genetic scaffolds within a single strain carry ARGs, the final *SRI* corresponds to the maximum score among all individually assessed elements.

The Integrated Risk Magnitude (R), ranging from 0 to $\sqrt{3}$, was derived as the Euclidean modulus of the risk vectors to reflect the convergence of phenotypic (PRI), genotypic (GRI), and spreading (SRI) hazards:

$$R=\sqrt{PRI^{2}+GRI_{norm}^{2}+SRI^{2}}$$

Based on the geometric distribution of this three-dimensional vector space, the health risk was stratified into four non-linear tiers: Low (R<0.5); Moderate (0.5≤R<1.0); High (1.0≤R<1.4); and Critical (R≥1.4). These unequal intervals are predicated on the biological significance of multidimensional risk accumulation. Specifically, the “Critical” threshold of 1.4 reflects the simultaneous maximization of at least two risk categories (e.g., $\sqrt{1.0^{2}+1.0^{2}+0^{2}}\approx 1.414$), representing the dangerous co-occurrence of pan-drug resistance alongside high-frequency mobility or severe pathogenicity. This stratification ensures that the most hazardous “convergent” phenotypes are prioritized for immediate clinical or environmental biocontainment.

## 3. Results

### 3.1. Phylogenomic Positioning and Virulence Homology

**Figure 1:**
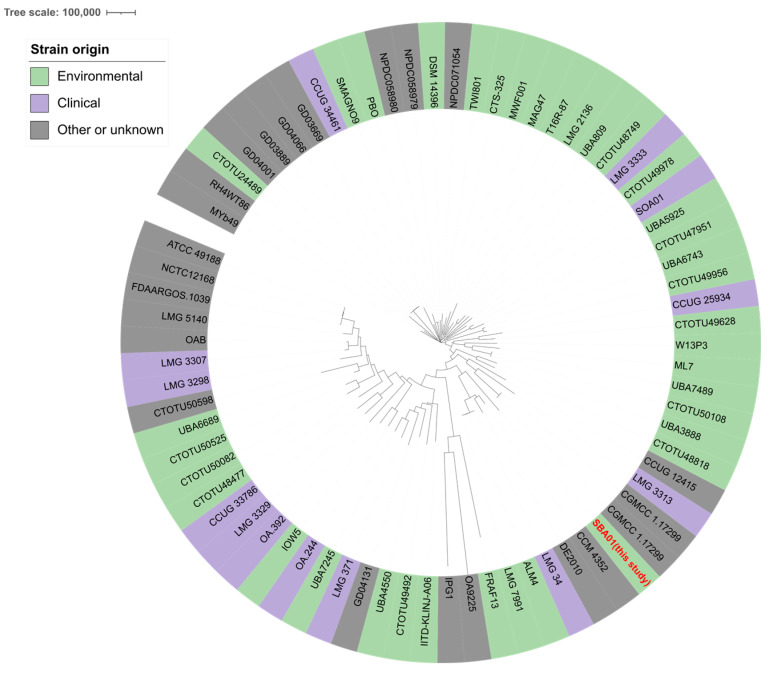
Phylogenomic reconstruction of *B. anthropi*. A Neighbor-Joining (NJ) tree was constructed based on global whole-genome single-nucleotide polymorphisms (SNPs) extracted from 72 *B. anthropi* genomes. Colored outer rings indicate isolation sources: green (environmental), purple (clinical), and grey (other/unknown). SBA01 (highlighted in red) clusters within a mixed-source clade, reflecting the ecological overlap and genetic exchange between environmental reservoirs and clinical settings.

Preliminary phenotypic and MALDI-TOF identification was rigorously corroborated by genomic taxonomy. Strain SBA01 exhibited an ANI of 97.71% relative to the *B. anthropi* ATCC 49188T genome, unequivocally confirming its species assignment. The assembly of *B. anthropi* SBA01 reached high contiguity, resolving all five plasmids as complete circular molecules (Table S8). The sequencing depth for these plasmids ranged from 112.5× to 134.4×, with a normalized coverage of approximately 1.1–1.3, ensuring high-fidelity consensus sequences. Phylogenomic reconstruction based on whole-genome SNPs characterized the internal population structure of the 72 *B. anthropi* isolates. The resulting topology demonstrated that these genomes do not strictly segregate out by isolation source. Evaluated by clustering distances, SBA01 localizes within a defined mixed-source lineage. Within this specific evolutionary branch, the environmental isolate SBA01 clusters alongside clinical isolates from diverse geographical origins (Figure 1). This topological interspersion indicates an absence of niche-restricted monophyletic divergence, suggesting a continuous molecular ecological overlap between open environmental reservoirs and clinical cohorts for this opportunistic pathogen.

Despite its environmental origin, genomic screening revealed that SBA01 harbors several structural homologs (exhibiting >85% amino acid sequence identity via BLASTp v2.17.0; Table S1) to classical determinants found in *Brucella melitensis*. These include an intact *bvrR/bvrS* two-component system (>90% identity) and the *lpx* operon. While these loci are associated with invasion in canonical pathogenic *Brucella*, in the broader context of the *Brucellaceae* family, they primarily govern fundamental outer membrane homeostasis and environmental stress tolerance. Furthermore, SBA01 possesses a complete *virB1*–*virB11* Type IV Secretion System (T4SS) and the associated *ricA* gene. Notably, topological analysis confirmed that this T4SS operon is localized on the conjugative helper plasmid (pSBA01-con) rather than the chromosome. This genomic architecture indicates that the T4SS in SBA01 acts primarily as a mating pair formation apparatus facilitating horizontal conjugation, rather than functioning as the canonical intracellular secretion machinery specialized for host interactions.

### 3.2. Multidrug Resistance Profile and Genetic Basis

Susceptibility profiling identified SBA01 as a multidrug-resistant isolate. The strain exhibited high-level resistance to clinically relevant β-lactams, particularly carbapenems (meropenem, 64 µg/mL; ertapenem, 4 µg/mL), and demonstrated resistance to colistin (8 µg/mL) (Table 1). Genomic analysis attributed this phenotype to specific molecular determinants (Table S2). Carbapenem resistance is driven by the metallo-β-lactamase gene *bla*_IMP-1_, which co-occurs with intrinsic AmpC β-lactamase gene *bla*_OCH-5_. Additionally, the genome harbors determinants conferring resistance to aminoglycosides (*aac*(*6′*)*-Ib*) and sulfonamides (*sul1*). Structural analysis showed that *sul1* is linked to quaternary ammonium compound resistance gene *qacEΔ1*, a typical feature of class 1 integrons. Consistency between genotypic resistome and phenotypic profiles confirmed that SBA01 is a reservoir of clinically significant resistance mechanisms.

### 3.3. Structure of the Plasmid-Mediated Resistance Mechanism

**Figure 2:**
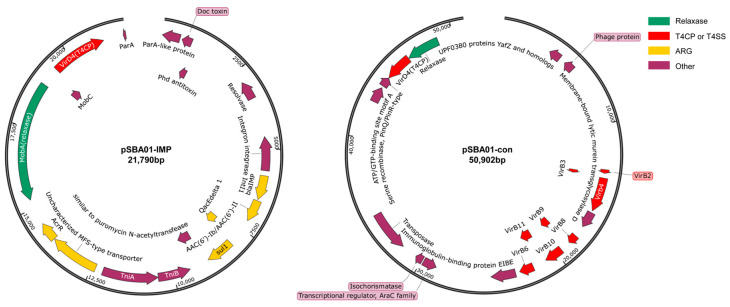
Bipartite mobilization architecture in *B. anthropi* SBA01. ((**Left**): The mobilizable resistance plasmid pSBA01-IMP (21,790 bp) carries the *bla*_IMP-1_ carbapenemase gene (yellow) and a minimal mobilization module (*mobA*, *mobC*), but lacks a functional conjugation bridge. (**Right**): The co-resident helper plasmid pSBA01-con (50,902 bp) encodes a complete Type IV secretion system (T4SS, *virB* operon, red), providing the distinct mating pair formation machinery required for *trans*-mobilization).

The multidrug resistance phenotype of SBA01 was primarily associated with the 21,790 bp plasmid pSBA01-IMP (Figure 2). Although this plasmid is mobilizable, it lacks a self-sufficient transfer region. Genomic analysis revealed that its dissemination was facilitated *in trans* by co-resident helper plasmid pSBA01-con (50,902 bp). This helper plasmid encodes a complete T4SS operon (*virB1*–*virB11*), which provides mating pair formation machinery. In this bipartite model, pSBA01-IMP likely employed its encoded relaxase to dock with the conjugative bridge established by pSBA01-con. Conjugation assays validated this uncoupled mobilization mechanism. The *bla*_IMP-1_ element transferred at a frequency of (4.2 ± 0.5) × 10^−6^ transconjugants per donor cell for the *K. pneumoniae* K19 recipient, whereas transfer to *E. coli* J53 occurred at a markedly lower frequency of (3.8 ± 1.2) × 10^−8^. No transfer was observed in DNase I-treated or uni-micro-microbial negative controls, empirically excluding natural transformation. Phenotypically, the transconjugants strictly mirrored the donor regarding *bla*_IMP-1_-associated carbapenem resistance profiles (Table 1). To delineate the segregationally dynamics post-conjugation, short-read sequencing was performed on the transconjugant TBK1. Mapping metrics unambiguously confirmed the co-transfer of the bipartite system, with both plasmids aligning perfectly to the donor reference templates (Figure S1). Notably, quantitative read-depth analysis revealed disparate maintenance trajectories (Table S8). Normalized against the recipient chromosomal depth (~200×), the target resistance plasmid pSBA01-IMP maintained a stable depth of 250–285× (estimated PCN ≥ 1), driven by experimental selection. Concurrently, the helper plasmid pSBA01-con was detected at a sub-chromosomal depth (~117.2×, PCN ≈ 0.58). This fractional PCN indicates active segregational loss of the helper plasmid within the transconjugant population during initial subculturing, a trajectory consistent with its transient mating pair formation function and the absence of an independent selection marker.

**Figure 3:**
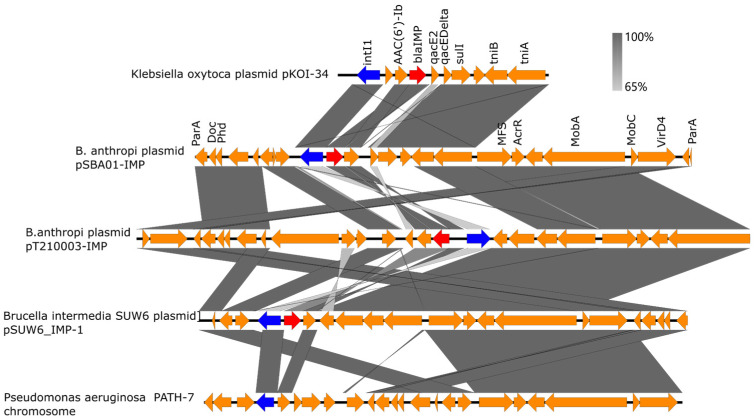
Cross-species dissemination of the *bla*_IMP-1_ integron array. (Linear comparative genomic analysis aligns the *bla*_IMP-1_-harboring region of pSBA01-IMP (second row) against diverse genetic contexts. Grey connecting bands indicate regions of high nucleotide identity (>99%). Third and forth row show extensive backbone conservation with plasmids from clinical *Brucella* isolates (pT210003-IMP, pSUW6_IMP-1), highlighting a conserved ancestral lineage. Top and Bottom rows demonstrate the capture of the identical *bla*_IMP-1_ integron array by high-priority pathogens (*Klebsiella oxytoca*, *Pseudomonas aeruginosa*), evidencing horizontal gene transfer across phylogenetic barriers. Arrows indicate open reading frames (ORFs): red (*bla* genes), blue (integrases), orange (other resistance/mobile elements)).

Plasmid stability was maintained through type II toxin–antitoxin (TA) system (*doc*/*phd*). This mechanism induces post-segregationally killing of plasmid-free daughter cells, promoting persistence of resistance determinants in the absence of antibiotic selection pressure. Structurally, pSBA01-IMP backbone shares extensive synteny with plasmids from clinical *Brucella* isolates (including *B. anthropi* T210003 and *B. intermedia* SUW6). Conservation of stability (TA system) and mobilization modules (*mobA*, *mobC*, *virD4*) suggests a shared plasmid lineage circulating within *Brucellaceae* (Figure 3, third and forth rows).

Notably, the genetic context of *bla*_IMP-1_ extends beyond *Brucella*. An identical integron array was identified on plasmid pKOI-34 of *Klebsiella oxytoca* and integrated into chromosome of *Pseudomonas aeruginosa* strain PATH-7 (Figure 3, top and bottom rows). This homology provides evidence that pSBA01-like elements serve as vectors for horizontal transfer of carbapenem resistance between environmental *Brucella* reservoirs and clinical pathogens, potentially facilitating chromosomal integration via transposition.

Subsequent targeted genomic screening of SBA01 identified chromosomal structural mutations primarily localized within two-component systems and efflux pump regulatory elements, notably *phoP*, *mexP*, and *nfxB* (Table S9). These intrinsic variations are traditionally associated with polycationic peptide resistance. Following conjugation, the resulting transconjugants (TBJ1, TBK1) exhibited significantly elevated colistin MICs (4–8 µg/mL) despite the absence of known mcr genes. Annotation of the co-transferred episomes revealed an array of envelope-modulating elements: the 50 kb conjugative helper plasmid encodes a membrane-bound lytic murein transglycosylase D (*MltD*) and an *AraC*-family transcriptional regulator, while the mobilizable plasmid carries the small multidrug resistance (SMR) efflux transporter qacEΔ1.

Additionally, bioinformatic analysis identified intact prophage elements integrated within the megaplasmid architecture (Table S3). While these prophage regions do not directly harbor antimicrobial resistance determinants, their stable integration further illustrates the highly plastic and recombinogenic nature of this genetic element, highlighting its role as a versatile platform for the continuous accretion of mobile DNA.

### 3.4. Multidimensional Quantitative Risk Index

Application of the quantitative risk framework classified SBA01 as a critical-level hazard (R = 1.63). The comprehensive calculation matrix, detailing the specific parameters, reference weights, and phenotypic/genomic inputs for each dimension, is provided in Table 2 and Table S7. PRI was 0.81, driven by resistance to high-priority agents; specifically, non-susceptibility to carbapenems and colistin limited therapeutic options. GRI reached its maximum value (1.0), defined by convergence of rank I determinants and *Brucella*-homologous virulence cluster. Importantly, this maximum GRI score does not imply that SBA01 exhibits the canonical intracellular pathogenesis of classical Brucella. Rather, it reflects the dangerous convergence of rank I antibiotic resistance determinants (blaIMP-1) with robust Brucellaceae-homologous resilience clusters (bvrR/S, lpx). Significantly, SRI highlighted discrepancy between prediction and experimental validation, whereas in silico mining failed to detect canonical transfer modules (predicted risk = 0), experimental results necessitated correction to maximum score (SRI = 1.0). Integration of these vectors yielded a Total Risk Magnitude (R) of 1.63 (surpassing the critical threshold of 1.4), indicating a high genomic risk potential within this evaluated framework.

Furthermore, a benchmarking analysis was performed against a susceptible wild-type *B. anthropi* reference (ATCC 49188T) and a clinical strain T210003 lacking virulence and highly similar to the drug-resistant gene of SBA01 As shown in the benchmarking results, the wild-type strain ATCC 49188T exhibited a R score of 0.48 (Low Risk), primarily driven by its intrinsic genomic background with zero acquired ARGs and no mobile potential (SRI = 0.3). Although the clinical strain T210003 carried acquired ARGs (GRI_raw = 27) and demonstrated moderate phenotypic resistance (PRI = 0.47), its total risk remained at 0.64 (Moderate Risk) due to the lack of high-tier virulence factors and restricted plasmid mobility.

## 4. Discussion

Identification of *bla*_IMP-1_-carrying *B. anthropi* in municipal wastewater-irrigated soil underscores evolving role of nonfermenting Gram-negative bacteria as underestimated reservoirs involved in environmental dissemination of carbapenem resistance. Although *B. anthropi* is typically regarded as a commensal soil organism or opportunistic pathogen of low virulence [7,9], these genomic findings reveal its potential to act as a resilient genetic vehicle for high-risk ARGs at the wastewater–soil interface. Specifically, the isolation of strain SBA01 demonstrated that environmental habitats under anthropogenic pressure have become assembly sites for multidrug-resistant genetic scaffolds. These genetic linkages, previously considered hallmarks of high-risk clinical Enterobacteriaceae, are now consolidated within resilient soil populations, effectively bridging gap between environmental resistomes and opportunistic human pathogens [30,31].

A pivotal finding in this study was genomic stability of *bla*_IMP-1_ within the 21 kb plasmid, maintained by type II TA system [32]. Bacterial persistence in soil environments requires metabolic efficiency, and maintenance of ARG-carrying plasmids typically imposes fitness cost [33]. However, identification of plasmid addiction system in SBA01 explains maintenance of multidrug-resistant phenotype even in absence of direct antibiotic selection pressure. This mechanism effectively locks resistance determinants within population, prevents plasmid curing, and facilitates vertical transmission. Consequently, *B. anthropi* populations in wastewater-affected soils may function as long-term genetic hubs for carbapenemases capable of surviving fluctuating environmental conditions that might otherwise eliminate less adapted plasmid hosts [34,35].

Beyond persistence, transmission of resistance from SBA01 to *Escherichia coli* and *Klebsiella pneumoniae* demonstrates its intrinsic capacity for HGT under prescribed conditions. Although small 21 kb plasmid harboring *bla*IMP-1 lacks complete conjugative transfer region, the sequencing results indicate that co-resident unclassified 50 kb plasmid functions as helper element, providing necessary mating pair formation machinery [36]. This mobilization strategy represents distinct dissemination paradigm compared with pandemic spread of bla_KPC_ and bla_NDM_, which is predominantly driven by large, self-transmissible broad-host-range plasmids belonging to IncF, IncX3, or IncC incompatibility groups [37]. Unlike the autonomous expansion typically observed with large Inc-type plasmids, the helper-dependent mobilization of *bla*_IMP-1_ in SBA01 suggests a more cryptic mode of resistance persistence. Under this framework, small non-conjugative plasmids leverage pre-existing, broad-host-range conjugative networks of environmental helper elements to traverse phylogenetic barriers. While in vitro assays empirically demonstrate the feasibility of such interspecies exchange, extrapolating definitive directionality within natural ecosystems requires a cautious interpretative lens. The discovery of near-identical mobile genetic elements (MGEs) across disparate taxa—including clinical *Klebsiella* and *Pseudomonas*—may signify evolutionary convergence or independent acquisitions from a shared environmental reservoir, rather than strict unidirectional dissemination originating from *B. anthropi*. Consequently, in the high-density microbial matrices of wastewater-irrigated soils, SBA01 is characterized as a resilient participant within a broader genetic network. In this environment, sub-inhibitory antimicrobial pressures and high cell densities likely facilitate the stochastic and bidirectional shuffling of resistance determinants between environmental opportunists and clinical lineages [5,37].

Beyond horizontal element exchange, the *mcr*-independent colistin resistance observed in SBA01 highlights complex chromosomal adaptations. The identified mutations in regulatory loci, such as *phoP* and *mexP/nfxB*, are established drivers of lipid A structural modifications and compensatory efflux pump overexpression in related Gram-negative species [38]. Furthermore, the functional acquisition of elevated colistin MICs in transconjugants—despite the absence of direct *mcr* determinants on the plasmids—indicates an intricate plasmid-host regulatory crosstalk. It is postulated that extrachromosomal elements form a non-canonical trans-activation network. Specifically, exogenous expression of the plasmid-encoded *MltD* enzyme and continuous *qacEΔ1* transporter activity likely perturb localized peptidoglycan homeostasis and alter transmembrane electrochemical gradients. These structural interventions may act as physical stimuli, triggering severe envelope stress responses (ESRs) in recipient cells. Upon activation, ESR pathways can bypass standard regulatory checkpoints, upregulating latent intrinsic resistance mechanisms. Additionally, the co-transferred *AraC*-family transcriptional regulator may interact with chromosomal promoters to activate endogenous efflux or envelope-remodeling operons. Although this combinatorial model integrates mechanical envelope stress with regulatory crosstalk, definitive validation requires targeted transcriptomic and mutagenesis investigations.

The convergence of these multifaceted resistance networks and active mobilization dynamics fundamentally drives the high computed risk index (R = 1.63) assigned to SBA01. To contextualize this mathematical modeling, the result was benchmarked against reference strains. The distinct divergence between the susceptible wild-type (*B. anthropi* ATCC 49188T, R = 0.48), the clinically isolated multidrug-resistant strain lacking high-tier virulence (T210003, R = 0.64), and SBA01 (R = 1.63) illustrates how the index captures the genomic risk escalation mediated by the convergence of mobile resistomes and resilience factors. Consequently, within this evaluation framework, SBA01 emerges as a high-priority genomic candidate for monitoring rather than a baseline environmental isolate. Crucially, its elevated risk profile is not defined by classical acute intracellular pathogenesis, but rather by its genomic potential to act as a resilient, mobile vehicle for high-priority carbapenemases. Surveillance paradigms could therefore benefit from expanding beyond traditional fecal indicators to monitor such opportunistic environmental hosts, which possess both the genetic markers for persistence at the soil–water interface and the conjugative machinery with the potential to transfer multidrug resistance across microbiomes [39].

Integrating these findings within a One Health framework, the isolation of SBA01 from municipal wastewater-irrigated soil signifies a potential entry point for resistance determinants to interface with the community. Equipped with intrinsic soil adaptability and structural genomic stability, this lineage possesses the genetic architecture necessary to persist in agricultural matrices, independent of continuous wastewater seeding or direct selective pressure. Given the extensive irrigation history (2006–2019) at the sampling site, such agricultural soils function as long-term environmental niches. However, while the *Brucellaceae* family exhibits robust environmental resilience, the soil–aerosol–human transmission axis [40] requires careful contextualization. The current screening yielded a localized, singular clonal occurrence of SBA01 rather than a widespread ecological distribution. Without direct quantitative bioaerosol data, extrapolating the mechanical agitation of these soils into occupational respiratory colonization events remains strictly theoretical. Therefore, rather than projecting an immediate epidemiological threat, the identification of SBA01 exposes critical gaps in prevailing environmental monitoring strategies [41]. Traditional regulatory frameworks are generally insufficient to detect cryptic resistance reservoirs sequestered within non-traditional environmental hosts. Broadening future surveillance to incorporate genomic-based biomonitoring at the wastewater–soil interface is essential to objectively assess whether these localized resistomes dissipate intrinsically or possess the capacity to evolve into broader public health threats.

Several empirical constraints characterize the current findings. The singular recovery of the *bla*_IMP-1_ carrying lineage (SBA01) from limited composite soil samples (*n* = 4) represents a highly localized event. This restricted sampling scale logically precludes extrapolations regarding its widespread environmental prevalence or long-term ecological dominance across broader agricultural matrices. Furthermore, while the genomic architecture indicates robust adaptability to soil environments, the proposed soil–aerosol–human transmission axis acts primarily as a theoretical risk framework. In the absence of targeted bioaerosol quantification, the actual occupational respiratory hazard and aerosolization potential of SBA01 remain to be empirically established. Methodologically, the primary isolation protocol relied on MacConkey agar supplemented with 2 mg/L meropenem. While effective for capturing high-risk clinical phenotypes, this stringent selective pressure intrinsically biases recovery toward robust Gram-negative isolates expressing high-level carbapenem resistance. Consequently, this approach introduces a systematic risk of omitting fastidious environmental hosts or atypical lineages that harbor sub-clinical, low-level carbapenemases (e.g., OXA-48-like variants), potentially underestimating the true breadth of the cryptic resistome within the soil matrix. Despite these constraints, this high-resolution genomic case study serves as a vital molecular baseline, highlighting the necessity of expanding current biomonitoring paradigms beyond conventional fecal indicators to capture cryptic multidrug-resistant vectors at the wastewater–soil interface.

## 5. Conclusions

This study provides a detailed genomic characterization of *B. anthropi* SBA01, illustrating a sophisticated dual-plasmid mechanism for the maintenance and dissemination of *bla*_IMP-1_ in wastewater-irrigated soil. The calculated Genotypic Risk Index (R = 1.63) identifies an elevated genomic risk potential driven by the integration of structural robustness and active mobilization potential. However, as a localized case study, these findings do not imply a generalized epidemiological crisis but rather highlight a critical gap in environmental monitoring. Traditional surveillance frameworks focusing on fecal indicators may overlook such cryptic resistance vehicles. Broadening genomic biomonitoring to include resilient environmental hosts at the wastewater–soil interface is essential for objectively assessing the real-world trajectory of these emerging resistomes.

## Figures and Tables

**Table 1:** Antimicrobial susceptibility profiles (MICs) of *B. anthropi* SBA01, recipients transconjugants. (TBJ1 and TBK1 are respectively the transferable conjugative plasmids of *Escherichia coli* J53 and Klebsiella pneumoniae K19. MICs are expressed in μg/mL. Bold values indicate resistance according to CLSI (2020) and EUCAST (2020) breakpoints. Abbreviations: MEM, meropenem; ETP, ertapenem; CT, colistin; CZA, ceftazidime-avibactam; TGC, tigecycline; CTX, cefotaxime; CIP, ciprofloxacin; AMK, amikacin).

Isolate ID	MIC (μg/mL)							
	MEM	ETP	CT	CZA	TGC	CTX	CIP	AMK
SBA01	**64**	**4**	**8**	**>8/4**	0.5	**>16**	**>2**	8
K19	≤0.12	0.5	0.5	0.5/4	0.5	**>16**	**1**	16
J53	0.016	0.004	0.25	0.125	0.25	0.125	<0.04	4
TBK1	**64**	**4**	**8**	**>8/4**	0.5	**>16**	**>2**	32
TBJ1	**64**	**2**	**4**	**>8/4**	≤0.25	**>16**	**>2**	8

**Table 2:** Quantitative Risk Assessment Matrix and Baseline Benchmarking of *B. anthropi* Strains.

Risk Dimension	Metrics & Weighting	ATCC 49188 [8]	T210003 [11]	SBA01 (This Study)
Phenotypic (PRI)	Non-susceptibility/Weighted Clinical Max	0	0.47	0.81
	Key Features	Wild-type (S)	CR-MDR	CR-PDR/Colistin-R
Genotypic (GRI)	WARG (Acquired Determinants)	0 (None acquired)	27	27
	WVF (Tier 1:5/Tier 2:1)	38 (3 T1/23 T2)	5 (0 T1/5 T2)	76 (5 T1/21 T2)
	GRInormnorm (GRIrawraw/100)	0.38	0.32	1 (103 > 100)
Spreading (SRI)	Max (Experimental, Bioinformatic)	0.3	0.3	1
	Mobile Potential	Intrinsic Chromosome	Non-conjugative Plasmid	Conjugative/Mating (+)
Integrated (R)		0.48	0.64	1.63
Risk Tier	Defined Scale [0, 1.73]	Low (R < 0.5)	Moderate (0.5 ≤ R < 1.0)	Critical (R ≥ 1.4)

## Data Availability

The genomic data presented in this study are openly available in the NCBI Sequence Read Archive (SRA) and GenBank databases under BioProject accession number PRJNA860020.

## Supplementary Materials

The following supporting information can be downloaded at: https://www.mdpi.com/article/10.3390/microorganisms14030688/s1, Figure S1: Comparative plasmid alignment between the donor strain and transconjugants; Table S1: Profile of virulence determinants in *B. anthropi* SBA01; Table S2: Antimicrobial resistance gene profile of *B. anthropi* SBA01; Table S3: Annotation of genes located within the prophage regions of *B. anthropi* SBA01; Table S4: The evolutionary tree involves information about the genome; Table S5: The classification of drug-resistant genes used in the GRI calculation; Table S6: The toxicity gene classification used in the GRI calculation; Table S7: Details for calculating the R indicator using SBA01; Table S8: Sequencing and assembly quality parameters for plasmids in *B. anthropi* SBA01 and its transconjugants; Table S9: The mutation sites of the colistin resistance-related genes were identified in *B. anthropi* SBA01, 49188, and T210003.

## Abbreviations

The following abbreviations are used in this manuscript:

WWTPs	Wastewater treatment plants
ARGs	Antibiotic resistance genes
HGT	Horizontal gene transfer
CLSI	Clinical and Laboratory Standards Institute
PRI	Phenotypic Risk Index
GRI	Genotypic Risk Index
SRI	Spreading Risk Index
T4SS	Type IV secretion system
TA	Toxin–antitoxin
