# Supplementary figures and images for: First Report and Comprehensive Risk Index of blaIMP-1-Harboring Brucella anthropi in Municipal Wastewater-Irrigated Soil
Source: Microorganisms. 2026 Mar 18;14(3):688. doi: 10.3390/microorganisms14030688 (PMC13029125; doi:10.3390/microorganisms14030688)

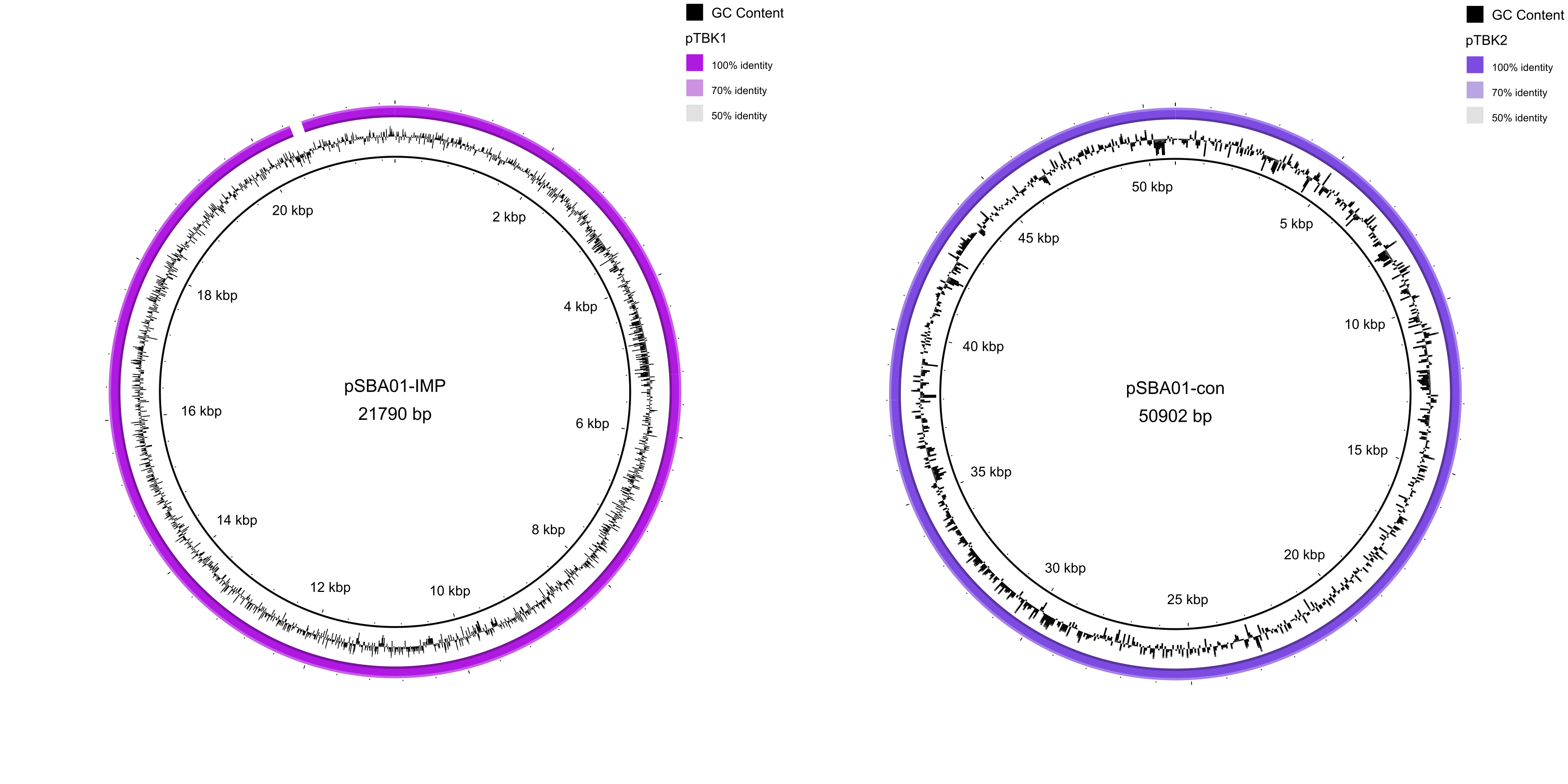

Supplement: Supplementary file 1 [file microorganisms-14-00688-s001.zip › FigureS1.tiff]
